# Minimum inhibitory concentrations of erythromycin and rifampin for *Rhodococcus equi* during the years 2007–2014

**DOI:** 10.1186/s13620-015-0051-4

**Published:** 2015-10-06

**Authors:** Caitriona S. Fenton, Thomas C. Buckley

**Affiliations:** University College Dublin, Belfield, Dublin, Ireland; Irish Equine Centre, Johnstown, Naas, Co. Kildare Ireland

## Abstract

**Background:**

*Rhodococcus equi* is a gram positive, intracellular pathogen of foals worldwide.

The aim of this study was to determine whether there was an increasing resistance occurring in *Rhodococcus equi* towards the antibiotics rifampin and erythromycin over a seven year period. The investigation was carried out with the use of E test strips (epsilometers) for rifampin and erythromycin in order to determine the Minimum Inhibitory Concentrations (MIC) values of *Rhodococcus equi* to these antibiotics.

**Results:**

The main results of this study found that the mean MICs were higher for erythromycin than for rifampin for every year analysed apart from 2008. The results highlight that 75 % (6/8) of the mean MICs for erythromycin were above the threshold of susceptibility of 0.5 μg/ml and one of the yearly mean MICs for rifampin (2008) was above the level of ≤ 1 μg/ml. Two soil samples analysed had high MIC values of 2 μg/ml and 3 μg/ml for rifampin and erythromycin respectively. These samples can be said to have acquired resistance as they are above 1 μg/ml.

**Conclusions:**

The significance of these findings is that *R. equi* is already a problematic pathogen to treat and if the bacteria keeps gaining resistance to these antibiotics at rate that has been shown over the last decade, then a new form of treatment will have to be introduced. Further research into the genomics of *Rhodococcus equi* will, in time, shed more light on possible alternatives such as vaccines or new, more effective antimicrobials.

## Background

*Rhodococcus equi* is a gram positive, intracellular soil saprophytic coccobacillus, classified in the order *Actinomycetales* [1]. First recognised as a pathogen of livestock by Magnusson in 1923, the bacteria was assigned the name *Corynebacterium equi*. It was only in 1977 that it was reclassified as *Rhodococcus equi*, (Rhodococcus means red-pigmented coccus) [2]. *Rhodococcus equi* is a worldwide pulmonary pathogen of foals from one to six months of age.

*R. equi* is an increasing pathogen of concern and is one of the leading causes of bronchopneumonia in foals. The prevalence and virulent nature of the disease has a major economic impact on the equine industry worldwide [3, 4]. The simple growth requirements of *R. equi* are met by herbivore manure and soil which make farms and stables an ideal breeding ground for the bacteria. The nature of this bacterium as a soil –dwelling organism coupled with intensification of equine breeding practices and management, makes this disease hard to control and prevent. *Rhodococcus equi* can be present in the environment of virtually all farms. However, some farms suffer from endemic clinical disease compared to sporadic and unrecognised clinical cases on others [4]. Foals, in particular, are most at risk of the disease due to a number of factors, including an undeveloped immune system and the drop in maternal antibodies [5].

There are a wide range of antimicrobials to which *Rhodococcus equi* is susceptible to in vitro.However, finding an antimicrobial that works in vivo is a challenge.

The recommended antibiotic dosage for *R. equi* infection has been described as being : 25 mg/kg of erythromycin orally every 8 or 12 h and for rifampin: 5 mg/kg orally every 12 h, or 10 mg/kg every 24 h for a duration of 4–9 weeks [6].

Although well tolerated in most foals, the combination therapy of erythromycin and rifampin can cause adverse reactions. The most common side effect is diarrhoea which is usually self-limiting. However, if the diarrhoea persists, it can lead to dehydration [7]. One of the most serious adverse reactions of these antimicrobials is severe hyperthermia and tachypnea, which can often result in death of the affected foal. These particular side effects are a significant cause of concern, especially for veterinarians in warmer climates[6].

The recent sequencing of the *R. equi* genome has revealed many genes that are resistant to antimicrobials, the concern would be that these genes, through the use of prophylactic or improper use of antimicrobials, could manifest over the coming years [6]. The nature of *R. equi* as a soil saprophyte means that it possesses many chromosomal resistance determinants that have evolved with the core genome. These determinants play a role in counteracting the action of naturally occurring antimicrobials. [8]. Resistance to rifampin has been shown to occur through a mutation in the ß subunit of rpoB gene [9]. The resistance mechanisms of macrolide resistance have not yet been determined on a molecular level. However, *R. equi* isolates that are resistant to either erythromycin, azithromycin or clarithromycin, are usually resistant to all three [8].

This study sought to determine whether there is a trend of resistance occurring for *Rhodococcus equi* to erythromycin and rifampin. This was carried out over a sample range dating from 2007 to 2014 to see if there was an increase in resistance.

## Methods

A total of 74 isolates, positively identified as *Rhodococcus equi* that were isolated from clinical samples submitted to the Irish Equine Centre between the years 2007–2014, were stored at −80 °C. The isolates were re-cultured using a pure colony for the inoculation of a fresh agar plate the MIC values were analysed. The MIC values of these isolates were analysed for both erythromycin and rifampin using gradient MIC strips (Liofilchem® MIC Test Strips). The test strips had an easily readable gradient of which the range was 0.016-256 μg/ml for both antibiotics. The strips created a clear zone of inhibition that was visible to the eye and the MIC was determined by establishing where the zone of inhibition began.

## Results

The results of the isolates analysed were grouped according to the year in which they were isolated. The MIC value was determined for each of the 74 isolates for both antibiotics. The range on each antibiotic strip was 0.016-256 μg/ml. The mean MIC values for each year can be observed in Table 1.

**Table 1:** Mean and ranges of the MIC concentration of *R. equi* to rifampin and erythromycin for each year (μg/ml)

Year of sample (number of samples)	Rifampin (range)	Erythromycin (range)
2014 (4)	0.300 (0.19 - 0.38 )	0.657 ( 0.38- 1.00 )
2013 (10)	0.345 ( 0.19 - 0.75 )	0.800 ( 0.75- 1.50 )
2012 (10)	0.308 ( 0.125-0.500 )	0.439 ( 0.25- 0.75 )
2011 (10)	0.408 ( 0.19- 0.75 )	0.514 ( 0.38-0.75)
2010 (10)	0.325 (0.125- 0.50 )	0.775 ( 0.50- 1.00 )
2009 (10)	0.689 ( 0.25- 2.00 )	1.125 ( 0.50- 3.00 )
2008 (10)	1.025 ( 0.50- 1.50 )	0.115 ( 0.047-0.19 )
2007 (10)	0.489 ( 0.125 - 1.50 )	1.225 ( 0.50- 2.00 )

The table shows that the mean MIC values vary significantly from year to year. However, they are still quite high. *Rhodococcus equi* is thought to be susceptible to rifampin at ≤ 1 μg/ml, and to erythromycin at ≤ 0.5 μg/ml. Therefore, according to these results, 75 % (6/8) of the mean MICs for erythromycin were above the threshold of susceptibility and one of the yearly mean MICs for rifampin (2008) was above the level of ≤ 1 μg/ml. The mean MIC concentrations for erythromycin were higher in nearly every year (exception 2008) compared to that of rifampin. Two isolates of *Rhodococcus equi* from the year 2009 were isolated from soil samples. The isolates had the highest MIC levels of all the 74 isolates, the MICs for the soils samples were 2 μg/ml for rifampin and 3 μg/ml for erythromycin. The samples were isolates from the Kildare region.

### Statistical Analysis (Table 2)

**Fig. 1:**
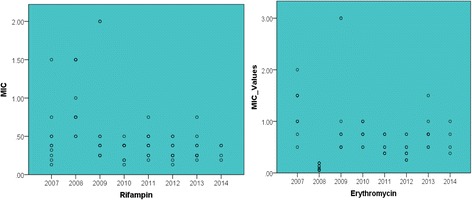
MIC Values for Rifampin and Erythromycin from 2007 to 2014 (μg/ml)

Figure 1 above graphically highlights the MIC values for each antibiotic throughout the sample period. There are outliers in 2009, these are soil samples. It can be hypothesised that these samples taken from the soil may have virulence plasmids which could be a reason for their resistance and therefore their high MIC values.

**Table 2:** Descriptive Statistics describing the Rifampin and Erythromycin MIC values measured yearly from 2007 to 2014

Rifampin MIC Values								
	2007	2008	2009	2010	2011	2012	2013	2014
Mean	0.49	1.03	0.69	0.33	0.41	0.31	0.35	0.30
Median	0.38	0.88	0.38	0.38	0.38	0.25	0.25	0.32
Variance	0.16	0.19	0.49	0.01	0.03	0.02	0.03	0.01
Std. Deviation	0.40	0.43	0.70	0.12	0.16	0.13	0.17	0.10
Minimum	0.13	0.50	0.25	0.13	0.19	0.13	0.19	0.19
Maximum	1.50	1.50	2.00	0.50	0.75	0.50	0.75	0.38
Range	1.37	1.00	1.75	0.37	0.56	0.37	0.56	0.19
Interqaurtile Range	0.33	0.81	0.63	0.19	0.25	0.18	0.16	0.18
Erythromycin MIC Values								
Mean	1.23	0.12	1.13	0.78	0.55	0.44	0.80	0.66
Median	1.25	0.11	0.75	0.75	0.50	0.38	0.75	0.63
Variance	0.20	0.00	1.00	0.03	0.03	0.04	0.08	0.08
Std. Deviation	0.45	0.06	1.00	0.18	0.18	0.19	0.28	0.28
Minimum	0.50	0.05	0.50	0.50	0.38	0.25	0.50	0.38
Maximum	2.00	0.19	3.00	1.00	0.75	0.75	1.50	1.00
Range	1.50	0.14	2.50	0.50	0.37	0.50	1.00	0.62
Interquartile Range	0.56	0.13	1.00	0.31	0.37	0.31	0.13	0.53

**Fig. 2:**
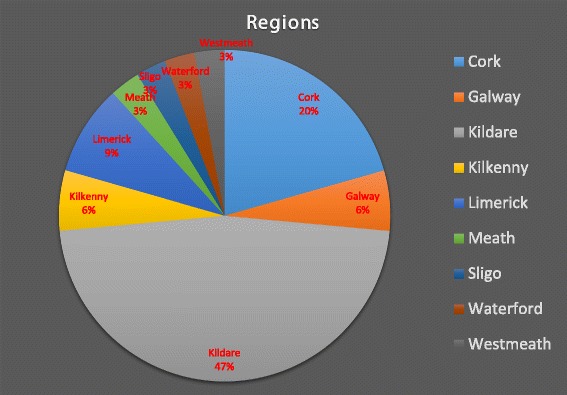
Samples analysed grouped by county of origin

Figure 2 above illustrates that the majority of the samples (47 %) originated from Co. Kildare and 20 % originating from Co. Cork. this is to be expected as most of the thoroughbred horses in Ireland are located in Kildare as well as the most amount of Equine veterinary hospitals, however further research would be warranted into a geographical link to resistant strains of the bacteria.

## Discussion

The aim of this project was to determine whether there was a trend of resistance occurring for *Rhodococcus equi* to the antimicrobials erythromycin and rifampin.

This analysis was carried out using isolates dating from 2007 to 2014 in order to establish if there was an increase in the Minimum Inhibitory Concentrations (MIC) over this timespan. This study was a follow on study from Buckley et al.,[10] who assessed the MIC values for *R. equi* isolates from pre- 2000 to 2006.

The 74 samples used in this study were analysed for their MIC values to erythromycin and rifampin using gradient strips for each antibiotic.

The MIC levels varied from year to year in this study. However, for erythromycin 75 % (6/8) of the mean MICs for all the years were above 0.5 μg/ml which indicates a trend of clinical resistance. According to Giguere et al., [7] the level of susceptibility for *R. equi* to erythromycin is ≤ 0.5 μg/ml . In the previous study conducted by Buckley et al., [10], only the mean MIC for the year 2006 was above this level at 0.583 μg/ml, indicating that the mean MIC levels may in fact be increasing with time. For rifampin in 2008, the mean MIC was above the susceptible level of 1 μg/ml. In the previous study, no levels for rifampin were above this level. Rifampin is always used in combination with another drug due to the fact that resistance to rifampin can occur quite quickly. Two isolates in this present study originated from soil samples and these samples had the highest MIC values of 2 μg/ml and 3 μg/ml for rifampin and erythromycin respectively. This is a worryingly high MIC value and may indicate that virulent *R. equi* is ubiquitous in the environment of farms. This indication warrants further research.

Buckley et al., [10] found that the MIC for rifampin increased from 1996 to 2006 and the MIC for erythromycin went from 0.258 μg/ml prior to the year 2000 to 0.583 μg/ml in 2006. The present study found that these MIC values have further increased in the last decade and the mean MIC values for 2014 were 0.657 μg/ml for erythromycin and 0.300 μg/ml for rifampin. This is a significant increase in MIC levels especially for a bacteria that is difficult to treat due to its intracellular nature and virulence plasmids.

The regions, where the isolates originated from, were also evaluated and the majority came from Kildare. This could be due to the fact that the majority of big stud farms are located in Co. Kildare. However, it could also point to a geographic link to *R. equi* virulence.

Data describing antimicrobial susceptibility for equine *Rhodococcus equi* isolates is scarce [11]. However, the incidence of resistant *R. equi* isolates is on the rise in recent years. A study by Boyen et al., [11] looked at antimicrobial resistance to *R. equi.* The study examined MIC values for *R. equi* to azithromycin, erythromycin , clarithromycin and rifampin. The results were similar to this present study in that of the 15 isolates tested for erythromycin, 10 were above 0.5 μg/ml, 5 were ≤ 1 μg/ml and for rifampin, one sample had an MIC of ≤ 1 μg/ml and 8 samples were ≤ 8 μg/ml. This suggests that resistance of *R. equi* to rifampin is increasing.

Takai et al., [9] described rifampin resistance back in 1997 and linked the resistance to the incorrect use of antibiotic therapy. Macrolide resistance seems to be more widespread than rifampin resistance. In this study, the mean MIC values were higher in every year (exception 2008) for erythromycin compared to rifampin. A study conducted by Burton [12], looked at clinical isolates from a Kentucky breeding farm to investigate macrolide and rifampin resistance after a screening program was initiated to detect subclinical *R. equi* pneumonia. The findings showed that seven years after the initiation of the program, which included prophylactic treatment of foals, the resistance to macrolides (including erythromycin) and rifampin, rose significantly. They found that 24 % of pre-treatment isolates showed resistance compared to a staggering 62 % of post-treatment isolates. This highlights the emergence of resistance over recent years and the need for correct diagnosis and treatment only when necessary.

This study highlights the acquired resistance of *R. equi* to the combination antibiotics of erythromycin and rifampin. Whilst the MIC values varied from year to year, this could be due to the relatively small sample size of 10 samples/year. Only four samples were analysed in 2014 due to the timing of the experiment. These were limitations of the study and further research should include a larger sample size.

The issue of antimicrobial resistance is a major concern for both human and animal public health. Due to the emergence of erythromycin and rifampin resistant *R. equi* isolates, alternative treatments must be researched further, as well as better management strategies for the issue of *Rhodococcus equi* infection. Better diagnostic methods should be researched further and avoidance of prophylactic treatments. Newer generation macrolides have been suggested as alternatives in combination with rifampin for the treatment of *Rhodococcus equi.* Of these, clarithromycin has been suggested as the best alternative for erythromycin. This was based on a study where the drug achieved satisfactory concentrations in the bronchoalveolar cells of foals and pulmonary epithelial lining fluid. This is a beneficial quality for a *R. equi* targeting antibiotic due to the intracellular activity of the bacterium [13]. Further research into the bioavailabity of antibiotics for the treatment of *Rhodococcus equi* is warranted.

## Conclusions

The overall conclusion from this study finds that the MIC values of erythromycin and rifampin to *Rhodococcus equi* have increased from levels before the year 2000 to 2014. This study highlighted that MIC levels for erythromycin are continuously higher than that of rifampin. However, the levels of rifampin, whilst not increasing from 2007 to 2014, are much higher than 2000–2006. The emergence of resistance to *Rhodococcus equi* further highlights the need for development of a suitable vaccine and for alternative ways to treat the disease. Environmental samples have been shown to have high MIC values to rifampin and erythromycin. This is worrying due to the nature of exposure to virulent *R. equi* from foals in their environment. Further research is needed to investigate the frequency and rate of acquired resistance of virulent *R. equi* isolates to erythromycin and rifampin.

## Abbreviations

*R. equi*: *Rhodococcus equi*
MIC: Minimum inhibitory concentration
Vap A: Virulence associated protein
